# The impact of spousal retirement on health—an empirical analysis based on CFPS

**DOI:** 10.3389/fmed.2024.1518936

**Published:** 2025-01-24

**Authors:** Ping Chen, Han Liang, Yufei Zhou, Guodong Zhu, Yu Peng, Jiayi Yao

**Affiliations:** ^1^Dong Fureng Economic and Social Development School, Wuhan University, Wuhan, China; ^2^Department of Economics, University of Southampton, Southampton, United Kingdom

**Keywords:** spouse retirement, health, regression discontinuity, mechanism analysis, mediating effect

## Abstract

**Introduction:**

Health impacts associated with spousal retirement vary significantly across countries and datasets, underscoring the need to examine this relationship within the Chinese context. This study aims to explore how spousal retirement affects individual health in China.

**Methods:**

Utilizing data from the China Family Panel Studies (CFPS) for the years 2014, 2016, 2018, and 2020, this research applies a fuzzy regression discontinuity approach to assess the effects of spousal retirement on health outcomes.

**Results:**

Findings indicate that a wife’s retirement positively impacts her husband’s mental health but negatively affects his physical health. Conversely, a husband’s retirement improves his wife’s self-rated health. Further analysis reveals that a husband’s retirement significantly enhances his wife’s marital satisfaction, contributing to longer sleep durations and, subsequently, better self-rated health.

**Discussion:**

This study highlights the nuanced effects of spousal retirement on individual health, with implications for understanding the marital and health dynamics within aging populations in China.

## Introduction

The dependence ratio among those aged 65 and above in China reached 22.5% at the end of 2023,^1^ almost double that of the senior population in 2012. The issue of rapid population aging in China has significantly strained the pension system. Simultaneously, the health issues brought about by the rapid aging of China’s population pose significant challenges to the medical insurance system. Retirement is closely linked to the health problems of middle-aged and elderly people. Exploring this issue will assist the government in implementing measures to address people’s health problems at this critical juncture of retirement, which will alleviate the pressure on medical insurance and the burden of social pension. According to the human capital theory, early retirement is unfavorable for the economic. Consequently, there is an imperative need to progressively adopt a retirement postponement policy. But in the meanwhile, the policy of delaying retirement has a bearing on the health level and interests of the general public. And health is a key factor in determining the pension cost of an elderly person. So, studying the effect of retirement on health can improve the awareness of policy makers about the importance of retirement, which will provide a basis for policy makers on how to formulate a retirement postponement policy. While several researchers have conducted study on this topic, there is comparatively less emphasis on exploring the effects of a spouse’s retirement on one’s health. Retirement does affect one’s health as well as the health of one’s spouse, and exploring the mechanisms involved can help people prevent some adverse health behaviors from occurring in advance, enhance the well-being of the middle-aged and elderly populations, and also provide lifestyle suggestions for them. In synthesizing, this paper explores the impact of spousal retirement on an individual’s health and the mechanisms involved. To address this, the study utilizes data from the Chinese Family Panel Studies (CFPS) to examine the influence of retirement behavior on the Chinese population.

There may be the following main innovations: First, this paper excludes the effect of individual retirement status on health by dividing the different retirement statuses of individuals, and further obtains the effect of spousal retirement on health. Second, this paper is more innovative in using individual satisfaction with marriage, spousal economic contribution, and spousal housework contribution to explore the mechanism of the effect of spousal retirement on individual health, which is a good addition to what is missing in the previous literature. Third, most of the literature on the measurement of health focuses on one or two dimensions, whereas this paper utilizes the CFPS to achieve a more comprehensive measure of the three dimensions of self-rated health, physical health, and mental health. Fourth, this paper uses four years of CFPS, including 2014, 2016, 2018, and 2020. Among them, the Family Relationships Database for 2020 will only be publicly released in January 2023, so we use this data earlier in our research.

The rest of this study is organized as follows. Section 2 reviews the relevant literature. Sections 3 and 4 introduces the data, variables, research design, and identification strategy. Sections 5, 6, 7, and 8 present the empirical results. Section 4 discusses the results and tests. Section 9 concludes the paper.

## Literature review

Previous research has proposed the notion of spillover effects of retirement on interpersonal relationships (1–5). Some research suggests that retirement can have spillover effects within the family (6), meaning that it affects not just individuals but also other family members, subsequent studies have provided support for this idea by explaining the external influences (7–9). In recent studies, it has been observed that retirement has implications for several aspects of individuals’ lives. Specifically, research has shown that retirement may influence family financial status (10), the division of labor within households (11), and marital status (12, 13). These factors may have an impact on one’s health, but seldom investigated on the impact on spousal health. Meanwhile, there is a limited number of articles that investigate the impact of a spouse’s retirement on health. Our article will provide a more complete discussion of the outcomes and mechanisms of the impact of spousal retirement on an individual’s health.

Some researchers have examined the health implications of spousal retirement and the underlying processes of impact, using data sourced from the United States, Japan, Europe, and the Netherlands. Nevertheless, the existing literature on the health implications of spousal retirement lacks a consensus due to variations in data sources and methodological approaches used across studies. Different aspects of health may provide disparate findings. One of these studies used data from the Health and Retirement Study (HRS) conducted in the United States to examine the influence of a spouse’s retirement duration on the manifestation of depressive symptoms subsequent to retirement (12). The study indicated that the retirement of spouses may positively affect males who have recently retired or have been retired for an extended period. The retirement of spouse might potentially have adverse effects on women who have not yet reached retirement age. However, using only one characteristic of depression as a measure of health is not sufficient here; our research will analyze this in multiple dimensions. In their study conducted in Japan, Bertoni and Brunello (10) discovered a significant correlation between the retirement of husbands and a decline in the mental well-being of their wives. This phenomenon, commonly referred to as “retired husband syndrome” primarily stems from the economic repercussions of the husband’s retirement, which subsequently heightens the financial burden on the family. Consequently, the wife experiences increased pressure, reduced sleep duration, and heightened levels of depression. In a study conducted by Mṻller and Shaikh (14), an analysis of SHARE data from 19 European nations revealed that the retirement of a spouse is associated with a rise in both the frequency and intensity of one’s own alcohol consumption. Moreover, there are significant gender differences in the impact of spouses’ retirement on health. In the US, men seem to be more likely than women to be negatively affected by their spouse’s retirement (16). This may be because men experience more physical health problems when their spouses retire, especially those who are no longer working when their spouses retire. However, in China, the husband’s mental health has improved after the wife’s retirement (17), which indicates that different cultural and social environments may lead to different health effects. In our paper, we explore other possible outcomes and mechanisms of the health effects of spousal retirement utilizing data from China. In their study, Atalay and Zhu (18) use data from the Household, Income and Labour Dynamics in Australia (HILDA) survey to investigate the empirical relationship between a woman’s retirement and her husband’s mental health. The findings of their research indicate that the retirement of a wife has a beneficial influence on the mental well-being of elderly men. Leckcivilize and McNamee (19) found that the retirement status of couples had no significant impact, which contrasts with the findings in other European countries, Japan, and Australia (10, 14, 18). Picchio and Van Ours conducted a study using panel data from the Netherlands, which also revealed that the retirement of a spouse had a positive impact on enhancing the well-being of their spouses (20). These articles use good methodologies to study the impact of spousal retirement on individual health, but the diversity of results they produce makes it difficult to refer to the papers’ conclusions to study the actual situation in China.

In recent years, there has been a growing scholarly interest in examining the health implications for Chinese individuals impacted by the retirement of their spouses. According to Chen’s study, the self-rated health and subjective well-being of husbands tend to be adversely impacted by their own retirement (21). Conversely, the retirement of husbands is more likely to have a beneficial effect on the physical and mental health of spouses, particularly among retired women. Using the same database, Wang found that husbands’ retirement did not affect their own self-rated health, but wives’ retirement significantly reduced husbands’ self-rated health because wives’ retirement raises intergenerational caregiving time, which in turn squeezes out spousal caregiving time, and therefore is detrimental to husbands’ health (22). First both studies are not the same database that we used for our study, and secondly in terms of the measurement of health, our study is probably more comprehensive. In Chinese dual-earner couples, the retirement of spouses has a significant direct and indirect negative impact on the individual’s cognitive health, and the retirement of husbands has a greater impact on the cognitive health of wives (23).

In addition, some studies provide a valuable contribution to the investigation of the mechanisms via which spousal retirement impacts health outcomes through influencing lifestyle behaviors. High levels of marriage quality can improve personal health (24, 25). The retirement of the husband has the potential to prompt the wife to engage in a reevaluation and renegotiation of the allocation of household responsibilities, resulting in marital discord and a decline in the woman’s overall health status (26) argues that he is constrained by the data to empirically test the possible mechanism of marital satisfaction. However, there are questionnaires related to marital satisfaction in the CFPS data, which are marital life satisfaction, satisfaction with financial contribution to spouse, and contribution to spouse’s housework. Therefore, we can use the CFPS data to test whether spousal retirement affects individual health through marital satisfaction. Yang et al. (27) suggested that there is gender heterogeneity in the impact of retirement on marital satisfaction among older adults. We will verify this point and extend it to whether there is gender heterogeneity in the effect on health.

This paper bridge the gap by using the CFPS to achieve a more comprehensive measure of the three dimensions of self-rated health, physical health, and mental health.

## Methodology

### Research design

#### Data

The data used for this study are sourced from the China Family Panel Studies (CFPS), a project conducted by the Institute of Social Science Survey (ISSS). The primary objective of the CFPS is to establish a comprehensive database for scholarly investigations and public policy analyses. By capturing individual, familial, and community-level data, the CFPS aims to depict the societal, economic, demographic, educational, and health-related transformations transpiring within China. This paper presents a study that incorporates data collected from four waves of surveys conducted in the years 2014, 2016, 2018, and 2020. The analysis utilizes two specific databases, namely the personal database and the family relationship database. CFPS survey data may not rule out possible bias in response rates for different populations, such as individuals with poor health who are less likely to answer questions, but in fact there are no studies showing such bias in CFPS data. Moreover, the focus of this paper is on the impact of retirement on health, focusing on capturing changes in health status, so it basically does not affect the empirical results of this paper. There are 89,513 samples.

Only individuals with an urban hukou were included in the sample. This decision was made because the urban *hukou* population is systematically covered by formal retirement systems, such as the Urban Employee Basic Pension Insurance (UEBPI), which provides structured retirement benefits directly relevant to our study. And in order to avoid the loss of part of the sample, it covers the rural groups where both spouses participate in the basic pension insurance for urban employees. In this paper, there is no difference in the retirement age between urban and rural *hukou*, and the retirement age of rural and urban *hukou* is the same for people of the same gender working in the same area and in the same unit. 40,867 samples of individuals and spouses who answered that they were not applicable, whose data were missing, and who did not participate in the endowment insurance for urban employees were deleted.

In order to mitigate the influence of age on the results, this paper employs a regression discontinuity design within a more limited retirement age range as specified in the policy (28). In accordance with the stipulations of the legal retirement age in China, the cutoff points are set at 60 for men and 50 for women, respectively. Consequently, only data pertaining to husbands aged between 50 and 70 and wives aged between 40 and 60 are retained to capture the pre-retirement and post-retirement phases. A total of 64,334 samples were deleted. This process yields a total of 11,120 pairs of spousal data, comprising 5,556 observations for male health and 5,564 observations for female health. Together with the deletion of some missing values for health data, there are 13,918 samples left.

We use the CFPS to achieve a more comprehensive measure of the three dimensions of self-rated health, physical health, and mental health. For self-rated health, participants were asked to evaluate their overall health status on a scale, which is a widely accepted method in health research. For physical health, we employed specific indicators such as chronic disease prevalence and functional limitations, which are standard measures in epidemiological studies. We referenced the Center for Epidemiological Studies Depression (CES-D) Scale for assessing mental health, which is a validated tool for measuring depressive symptoms.

#### Variables

##### Dependent variables

The dependent variables used in this study are the health status of the couple, as measured by the self-rated health, physical health and mental health, these multidimensional empirical investigations pertaining to self-rated health implications associated with retirement are the focus of this study. We used the total score of the Center for Epidemiologic Studies Depression (CES-D) scale as a proxy for mental health. To meet comparability, CES-D20 of CFPS2016, CFPS2018, and CFPS2020 were used as representative variables of mental health. A comprehensive analysis of the questionnaire items for CES-D8 and CES-D20 is provided in Appendix Table A1.

##### Running variables and treatment variables

The primary treatment variable is the retirement status of the spouse. This variable is represented as a dummy variable, taking the value of 1 if the running variable exceeds the specified cutoff point, where the running variable in our fuzzy regression discontinuity design is the age at retirement. The cutoff point is defined by the official retirement age in China, which is 60 years for males and 55 years for females. This cutoff serves as the threshold for analyzing the causal impact of retirement on spousal health outcomes.

##### Control variables

As noted above, variables as educational attainment, number of children, and *hukou* are all potential determinants that might significantly impact an individual health outcome. Hence, the present study has chosen these characteristics as control variables.

Furthermore, this study also identified the mediating variables. The exact variables are depicted in Appendix Table A2.

#### Descriptive statistics

According to Table 1 and Appendix Table A3, the retirement of the wife positively influences the mental health of the husband and vice versa but have a negative effect on their self-rated health and physical health. The husband’s retirement behavior has a positive impact on the wife’s mental health, but it also has a certain inhibitory effect on the wife’s self-rated health and physical health.

**Table 1:** Descriptive statistics of the male sample.

Variable types	Variables	(1)	(2)	(3)	(4)	(5)	(6)	(7)	(8)	(9)	(10)
		Wife unretired					Wife retired				
		Size	Average	SD	Min	Max	Size	Average	SD	Min	Max
Dependent variables	Self-rated health	4,064	3.088	1.102	1	5	1,500	3.183	1.065	1	5
	Physical health	4,064	0.188	0.391	0	1	1,500	0.236	0.425	0	1
	Mental health	2,848	30.315	7.288	20	70	962	29.008	6.580	20	70
Control variables	Personal retirement status	4,064	0.0662	0.249	0	1	1,500	0.352	0.478	0	1
	Age	4,064	54.980	3.946	50	70	1,500	57.933	3.889	50	70
	Spouse age	4,064	52.492	4.329	40	60	1,500	55.851	3.136	47	60
	*Hukou*	4,064	0.990	0.389	0	2	1,500	1.043	0.255	0	2
	Education	4,006	3.396	1.266	1	9	1,484	3.543	1.035	1	7
	Quantity of children	4,064	1.304	0.725	0	9	1,500	0.999	0.537	0	5
Mediating variables	Satisfaction of marital life	2,978	4.615	0.740	1	5	1,082	4.671	0.674	1	5
	Satisfaction with the spouse’s economic contribution	2,978	4.398	0.946	1	5	1,082	4.549	0.781	1	5
	Satisfaction with the spouse’s household contribution	2,978	4.530	0.828	1	5	1,082	4.630	0.745	1	5
	The proportion of housework	4,038	0.323	0.251	0	1	1,498	0.308	0.231	0	1
	The time spend watching TV or movies	4,064	13.341	11.76	0	140	1,500	16.040	12.40	0	70
	Dinner times with family	4,064	5.881	2.091	0	7	1,500	6.232	1.788	0	7
	Sleep time	4,058	7.316	1.297	2	18	1,500	7.258	1.268	2	12
	The number of times you exercise	4,064	4.000	3.421	0	30	1,500	4.504	3.233	0	21

By comparing the two situations, we found that there is a certain heterogeneity in the effects of a spouse’s retirement on health outcomes. This heterogeneity may be interpreted as a manifestation of gender-based disparities in the influence of a spouse’s retirement on an individual’s physical health. Meanwhile, we find that there is generally no heterogeneity in the mechanism variables of spouse retirement for individuals.

### Identification strategy

#### Benchmark model

The primary focus of this research is to the impact of spouse retirement on individual health. However, one’s own retirement also has an influence on his/her health. The exclusion of one’s own retirement may lead to omitted variable bias. Moreover, in most of the scholarly research, the individual’s retirement is considered the endogenous variable when investigating the influence of spousal retirement on an individual health. Hence, the commonly used least squares model may be expressed as follows.


$$H_{i}=\theta_{0}+\theta_{1}R_{i}+\theta_{2}R_{i}^{p}+\theta_{3}X_{i}+\varepsilon_{i}\text{,}$$

where $H_{i}$ denotes the health level, $R_{i}$ is the dummy for one’s own retirement which is 1 if he or she retires and 0 otherwise. $R_{i}^{p}$ is the dummy for spouse’s retirement which is 1 if one’s spouse retires and 0 otherwise. $X_{i}$ denotes the control variables, and $\varepsilon_{i}$ is the random error term.

Nevertheless, this baseline model exhibits significant endogeneity. The relationship between health and retirement is characterized by a reverse causal effect, wherein health can influence decisions regarding retirement and the retirement choices of one’s spouse. Notably, unexpected changes in health can have a significant impact on retirement behavior. Additionally, it is possible that there is an omitted variable issue present in the analysis. Personal endowments and preferences may affect both retirement decisions and health outcomes, leading to estimation bias.

This study employs the health effect model of spouse retirement and personal retirement as proposed by these studies (14, 15). The two-stage least squares (2SLS) method is used to identify the causal effect of spousal retirement on individual health outcomes.

The legal retirement age is denoted by the variables $C_{i}$ and $C_{i}^{p}$ , where the retirement age for males is set at 60 years and for women is at 50 years. The instrumental variables $D_{i}$ and $D_{i}^{p}$ are derived from the difference between age , $\mathrm{ag}e_{i}^{p}$ and the legal retirement ages $C_{i}$ , $C_{i}^{p}$ . The instrumental variables are defined as follows:


$$D_{i}^{\left(p\right)}=f\left(x\right)=\{\begin{array}{c} 0,\mathrm{if}\;s_{i}^{\left(p\right)}<0\\ 1,\mathrm{if}\;s_{i}^{\left(p\right)}\geq 0 \end{array}\text{,}$$

where


$$s_{i}^{\left(p\right)}=\mathrm{ag}e_{i}^{\left(p\right)}-C_{i}^{\left(p\right)}\text{.}$$

**Figure 1:**
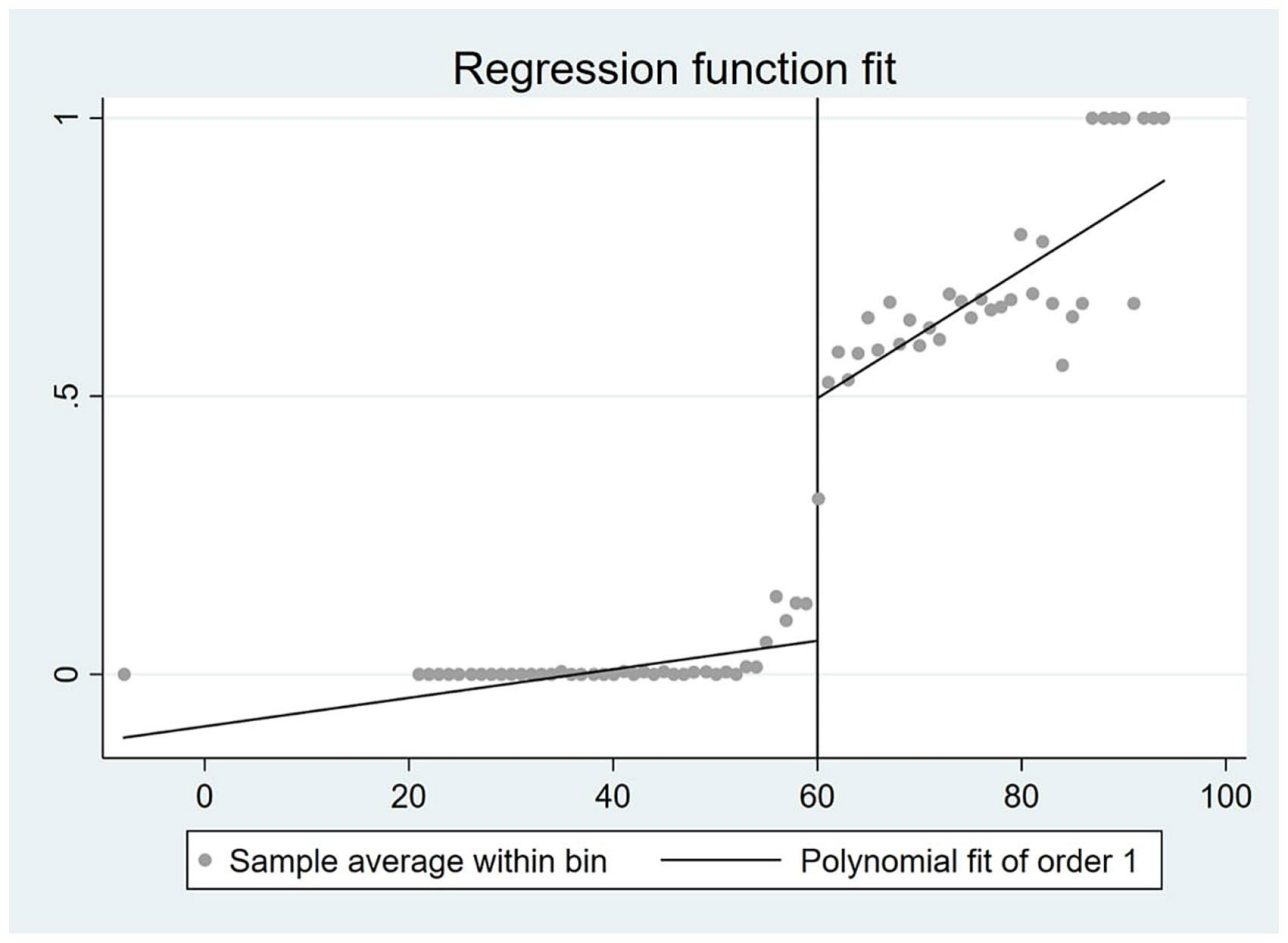
The correlation between age and the likelihood of retirement among males.

**Figure 2:**
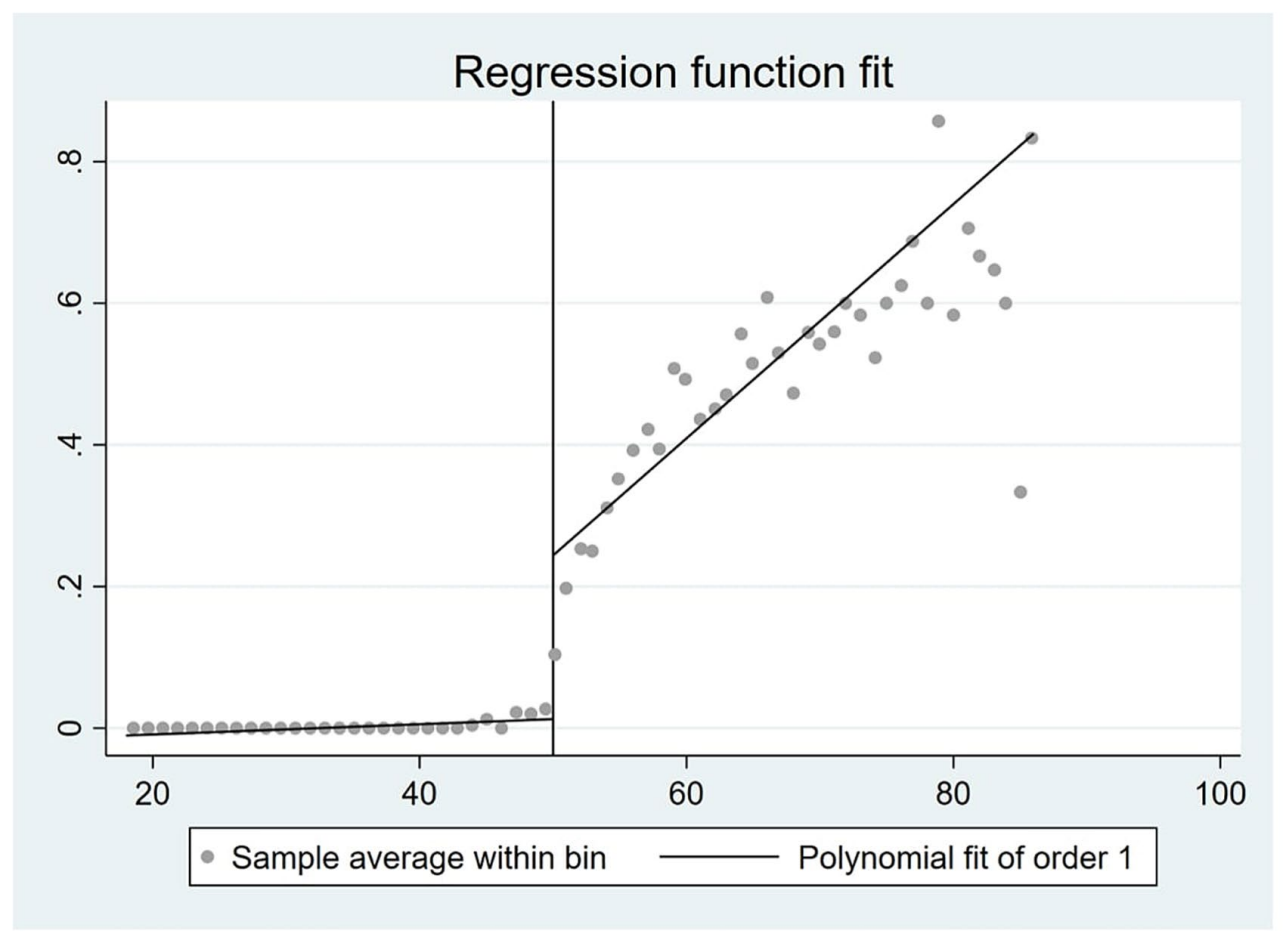
The correlation between age and the likelihood of retirement among females.

In the first stage, we regress one’s own retirement and the spouse retirement on the respective instrumental variables.


$$R_{i}=\alpha_{0}+\alpha_{1}D_{i}+\alpha_{2}s_{i}+\alpha_{3}D_{i}s_{i}+\alpha_{4}X_{i}+\varepsilon_{i0}\text{,}$$

$$R_{i}^{p}=\beta_{0}+\beta_{1}D_{i}^{p}+\beta_{2}s_{i}^{p}+\beta_{3}D_{i}^{p}s_{i}^{p}+\beta_{4}X_{i}^{p}+\varepsilon_{i1}\text{,}$$

where $D_{i}$ , $s_{i}$ , $D_{i}^{p}$ and $s_{i}^{p}$ are strategically constructed to exhibit different age trends on either side of the statutory retirement age, while also aligning with the graphical representations depicted in Figures 1, 2.

The regression in the second stage on


$$H_{i}$$

$$\begin{array}{l} H_{i}=\gamma_{0}+\gamma_{1}{\hat{R}}_{l}+\gamma_{2}s_{i}+\gamma_{3}D_{i}s_{i}+\gamma_{4}\hat{R_{l}^{p}}\\ \;+\gamma_{5}s_{i}^{p}+\gamma_{6}D_{i}^{p}s_{i}^{p}+\gamma_{7}X_{i}+\varepsilon_{i2}\text{,} \end{array}$$

where $H_{i}$ denotes individual health; $R_{i}$ and $R_{i}^{p}$ denote the retirement status of the individual and the spouse respectively; $X_{i}$ denotes the control variables (*hukou*, education, number of children). The coefficient $\gamma_{4}$ represents the impact of a spouse’s retirement on their individual health, while $\gamma_{1}$ denotes the impact of an individual’s own retirement on their individual health.

### Fixed individual’s retirement status

To control for the potential impact of individual retirement status and investigate the health implications of spouse retirement regarding heterogeneity in individual retirement, we conducted an additional analysis to assess the health impacts of spouse retirement given fixed individual retirement status.


$$R_{i}^{p}=\theta_{0}+\theta_{1}D_{i}^{p}+\theta_{2}s_{i}^{p}+\theta_{3}D_{i}^{p}s_{i}^{p}+\theta_{4}Z_{i}+\varepsilon_{i3}\text{,}$$

$$H_{i}=\delta_{0}+c\hat{R_{l}^{p}}+\delta_{2}s_{i}^{p}+\delta_{3}D_{i}^{p}s_{i}^{p}+\delta_{4}Z_{i}+\varepsilon_{i4}\text{,}$$

where $H_{i}$ denotes individual health; $R_{i}^{p}$ denotes the retirement status of the spouse; $D_{i}^{p}$ denotes the instrumental variable; $Z_{i}$ denotes the control variable (*hukou*, education, number of children, the difference between the age of the individual and the mandatory retirement age and its square). c measures the impact of the spouse’s retirement on individual health.

### Bootstrap test for intermediate effects

This paper discusses the mediation effect and applies the Bootstrap program in Stata to investigate the mediation effect of spouse retirement on individual health.


$$M=\varphi_{0}+a\hat{R_{l}^{p}}+\varphi_{2}s_{i}^{p}+\varphi_{3}D_{i}^{p}s_{i}^{p}+\varphi_{4}Z_{i}+\varepsilon_{i5}\text{,}$$

$$H_{i}=\omega_{0}+c^{\prime }\hat{R_{l}^{p}}+bM+\omega_{2}s_{i}^{p}+\omega_{3}D_{i}^{p}s_{i}^{p}+\omega_{4}Z_{i}+\varepsilon_{i6}\text{,}$$

where M is the mediating variable. The determination of a mediation effect necessitates the coefficients a , b , c , and $c^{\prime }$ as derived from Equations ((7, (8, (9).

The conditions for determining mediation effects can be categorized as follows: (1) when the coefficients a , b , and c are significant, but $c^{\prime }$ is not, only the mediation effect is observed; (2) when all coefficients a , b , c , and $c^{\prime }$ are significant, and ab and $c^{\prime }$ have the same sign, there is a partial mediating effect; (3) when c is significant, but at least one of the coefficients a and b is not significant, further analysis using the Bootstrap method is required to ascertain the presence of mediation effect.

## Results

### The impact of retirement policy on retirement behavior

In order to conduct a more comprehensive analysis of the impact of spousal retirement on the individual health, two distinct outcomes were presented: one with or without the inclusion of control variables.

Table 2 presents the outcomes of the initial stage regression analysis conducted on the male sample using the Two-Stage Least Squares (2SLS) method. Columns (1) and (2) present the results without considering the control variables, whereas columns (3) and (4) include these control variables.

**Table 2:** Results of two-stage least squares stage I estimation for the male sample.

Male	(1)	(2)	(3)	(4)
	Personal retirement	Wife retired	Personal retirement	Wife retired
Whether the individual exceeds the legal retirement age $D_{i}$	0.239*** (11.88)	-	0.236*** (11.79)	-
Whether the spouse exceeds the legal retirement age $D_{i}^{p}$	-	0.126*** (8.79)	-	0.124*** (8.47)
*Hukou*	-	-	0.033*** (4.15)	0.017*** (3.37)
Education	-	-	0.012*** (2.49)	0.105*** (8.34)
Quantity of children	-	-	−0.051*** (−9.28)	−0.094*** (−12.95)
The difference between the age and the legal retirement age	0.033*** (9.28)	-	0.033*** (9.14)	-
$D_{i}$ * (Difference between age and legal retirement age)	0.028*** (4.02)	-	0.029*** (4.23)	-
The difference between the spouse’s age and the legal retirement age	-	0.126*** (8.79)	-	0.021*** (14.71)
$D_{i}^{p}$ * (Difference between spouse age and legal retirement age)	-	0.021*** (15.71)	-	0.025*** (8.76)
Constant	0.177*** (13.24)	0.064*** (4.77)	0.189*** (10.90)	0.088*** (3.99)
Sample capacity	5,564	5,564	5,450	5,450
*F* value	212.377	324.043	152.371	205.528

***, **, and * represent significant at the significance levels of 0.01, 0.05, and 0.1, respectively. In parentheses are robust standard errors, unless otherwise specified, the same below.

The analysis of Table 2 reveals that the retirement policy exerts a substantial influence on the retirement behavior of both partners, irrespective of the inclusion or exclusion of the control variable. Upon reaching the legal retirement age, the likelihood of retirement for women increases by around 12% at a significance level of 1%. Conversely, men experience a retirement likelihood increase of 24% at a significance level of 1%, which is double that of women. The driving variable has a significant positive effect on retirement, indicating the effectiveness of the instrumental variables. Meanwhile, it is observed that upon reaching the legal retirement age, the rate of retirement for males exhibits a greater change in comparison to that of females. This discrepancy can be attributed to the fact that the legal retirement age for certain female workers is not 50 years old, but rather 55 years old.

By examining the coefficient of the difference between an individual’s age and the mandatory retirement age, as well as the disparity between age and retirement age in conjunction with the interaction term of instrumental variables, there exists a positive correlation between an individual’s age and the likelihood of retirement. Furthermore, it can be observed that as an individual’s age increases, the rate at which the likelihood of retirement increases also accelerates. These findings align closely with the outcomes depicted in Figures 1, 2.

Control variables such as *hukou* and educational attainment have been found to exert a considerable influence on individual retirement decisions. Moreover, an increase in the number of children is associated with a decreased likelihood of parental retirement.

The 1st stage regression outcomes for the female subsample exhibit similar magnitudes and significance levels for the own correlation coefficient as those observed in columns (2) and (4) of Table 2. Similarly, the magnitude and significance of the husband correlation coefficient align with the results in column (1) and (3) of Table 2. Consequently, these findings will not be reiterated here, and further details can be found in Appendix Table A4. The comprehensive sample’s first stage estimation results for 2SLS are presented in Appendix Table A5.

Additionally, the *F*-values of the first stage regression reported in the last column of Table 2, Appendix Tables A4, A5 are all far greater than 10, indicating the absence of any potential weak instrument bias.

### The impact of spouse retirement on individual health

The results of the two-stage least squares stage II estimation for the full sample for the full sample are displayed in Appendix Table A6. Columns (1), (2), and (3) represent the estimates without the inclusion of control variables, whereas columns (4), (5), and (6) include the control variables. The retirement of a spouse, regardless of the consideration of instrumental variables, has a significant detrimental impact on an individual’s physical health. This significance is consistently maintained at a level above 5%. The outcomes pertaining to mental health exhibit variability contingent upon the inclusion of control variables. The existing body of evidence suggests that the observed phenomenon can be explained by the significant gender variability in the association between the retirement of a spouse and the health outcomes of both the spouse and the individual (29). Hence, this study draws upon prior empirical research, specifically examining samples of both males and females, and doing comparative analyses with relevant previous studies.

#### Effect of wife’s retirement on husband’s health

**Figure 3:**
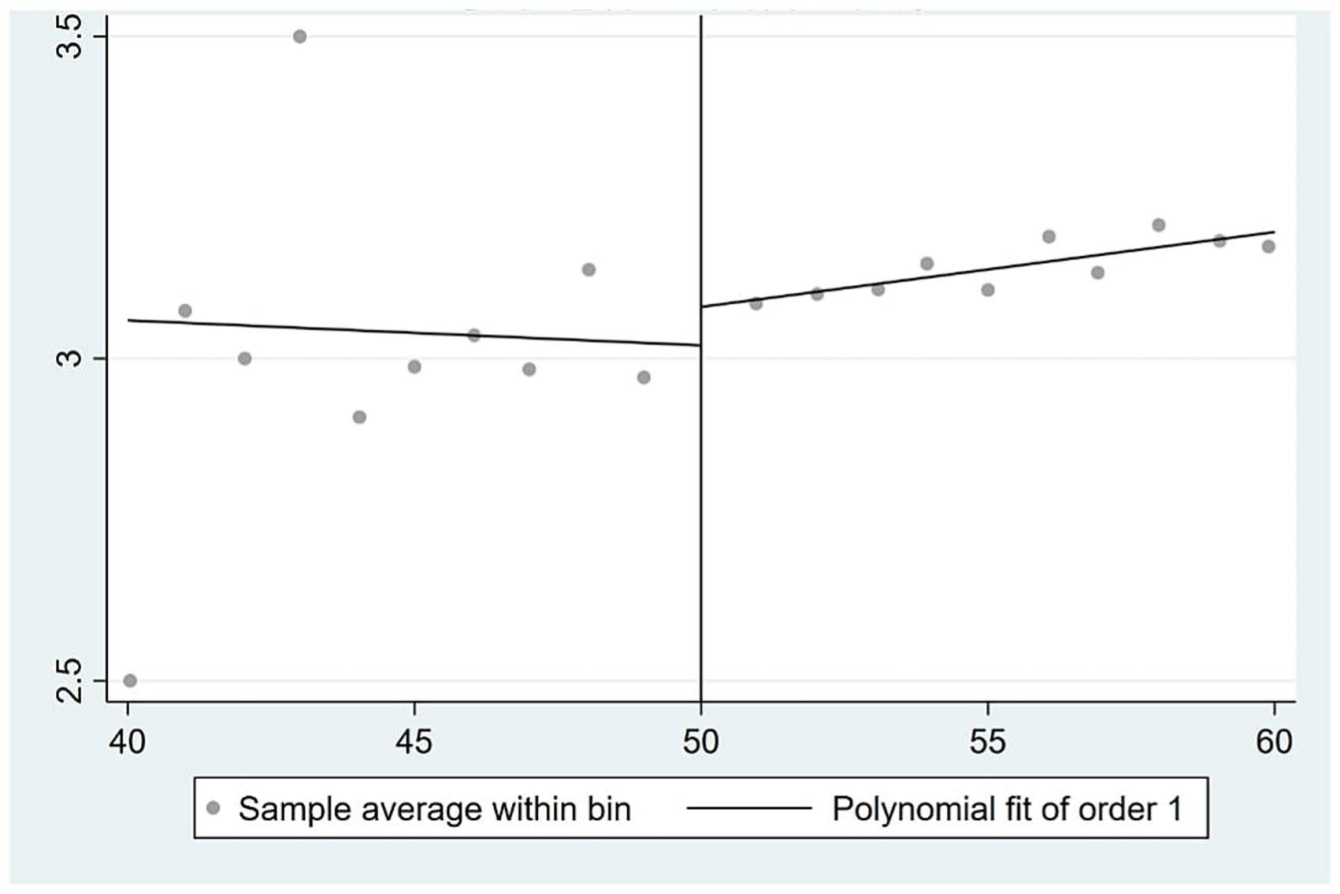
Effect of wife’s retirement on husband’s self-rated health.

**Figure 4:**
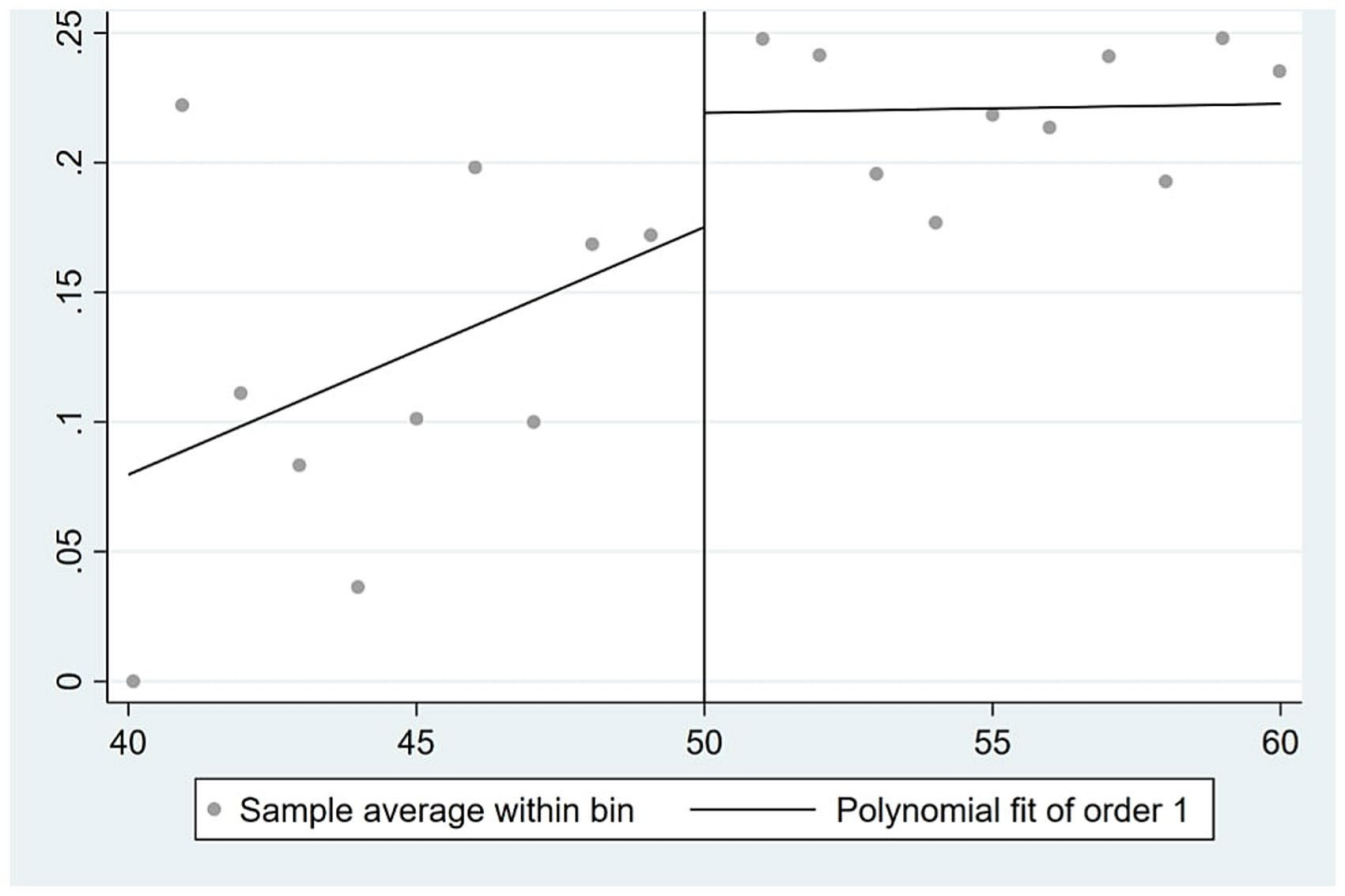
Effect of wife’s retirement on husband’s physical health.

**Figure 5:**
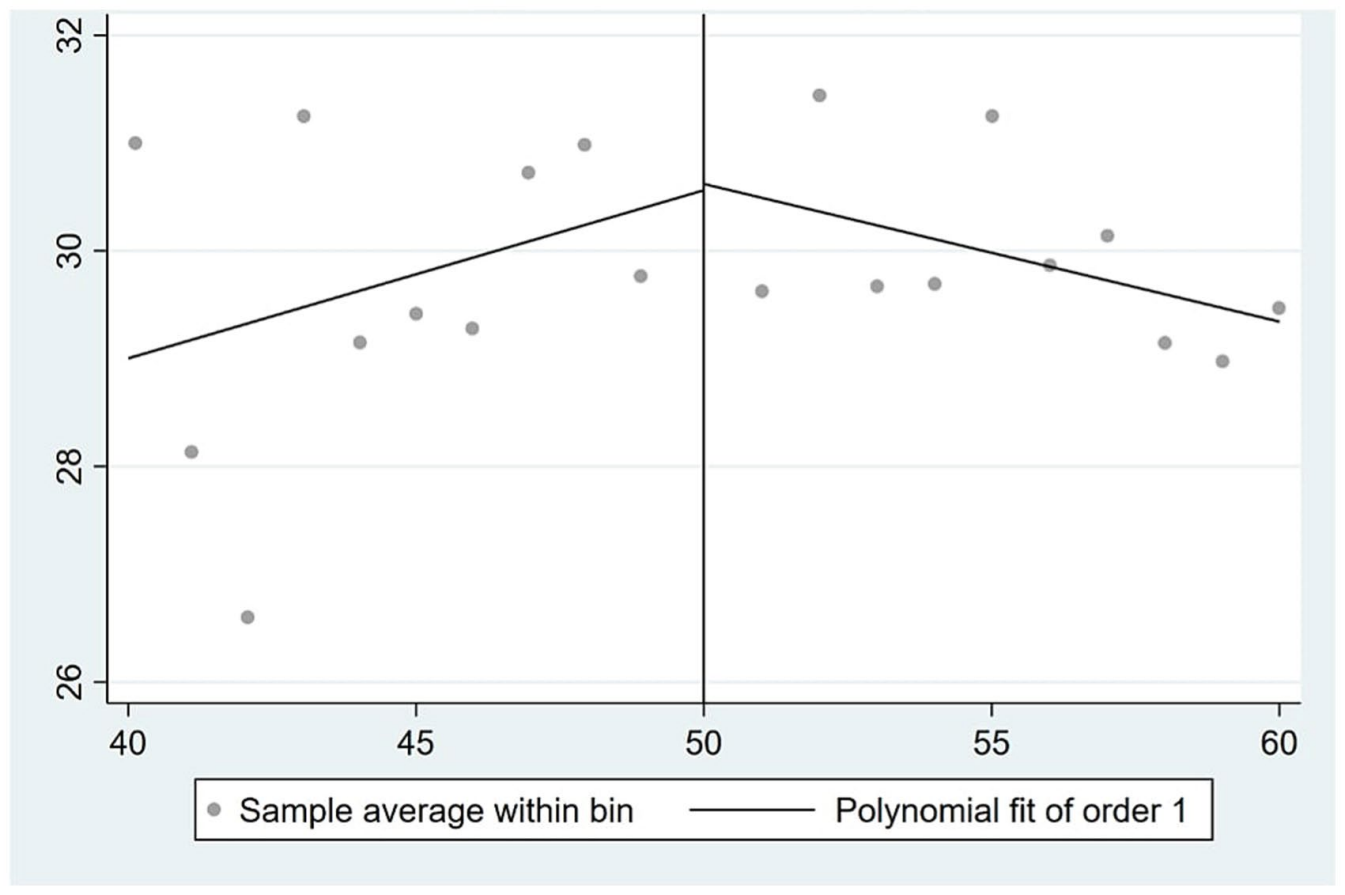
Effect of wife’s retirement on husband’s mental health.

Figures 3–5 illustrate the correlations between the husband’s self-rated health, physical health, and mental health, respectively, and the age of the wife. The impact of a wife’s retirement on her husband’s physical health is readily apparent, suggesting that the husband’s physical health condition is not positively affected by the wife’s retirement. Nevertheless, the impact on the husband’s self-rated health and mental health is rather limited.

In this paper, the 2SLS is used to further analyze the impact of wife’s retirement on husband’s health. These results are presented in Appendix Table A7. Columns (1), (2), and (3) do not account for control variables, while columns (4), (5), and (6) include control variables. The analysis of self-rated health indicates that wife’s retirement has a positive impact on the husband’s self-rated health, as evidenced by the findings in columns (1) and (4). This effect remains consistent regardless of the inclusion of control variables. The coefficient is below 0.2. According to the findings shown in columns (2) and (5), it can be observed that the retirement of the wife has a statistically significant adverse effect on the physical health of the husband, with a significance level of 5%. In relation to mental health, the findings in column (3) suggest that the retirement of spouses has a statistically significant positive impact on the mental health of the husband, with a significance level of 5%. Based on the findings shown in column (6), it is evident that the retirement of the wife has a statistically significant impact on reducing the CES-D depression quantification scale score by 7.315 units, with a significance level of 1%. This effect holds true even when controlling for other variables. In conclusion, the wife’s retirement had no significant effect on the husband’s self-rated health, but significantly reduced the husband’s physical health level and significantly increased the husband’s self-rated health level.

#### Effect of husband’s retirement on wife’s health

**Figure 6:**
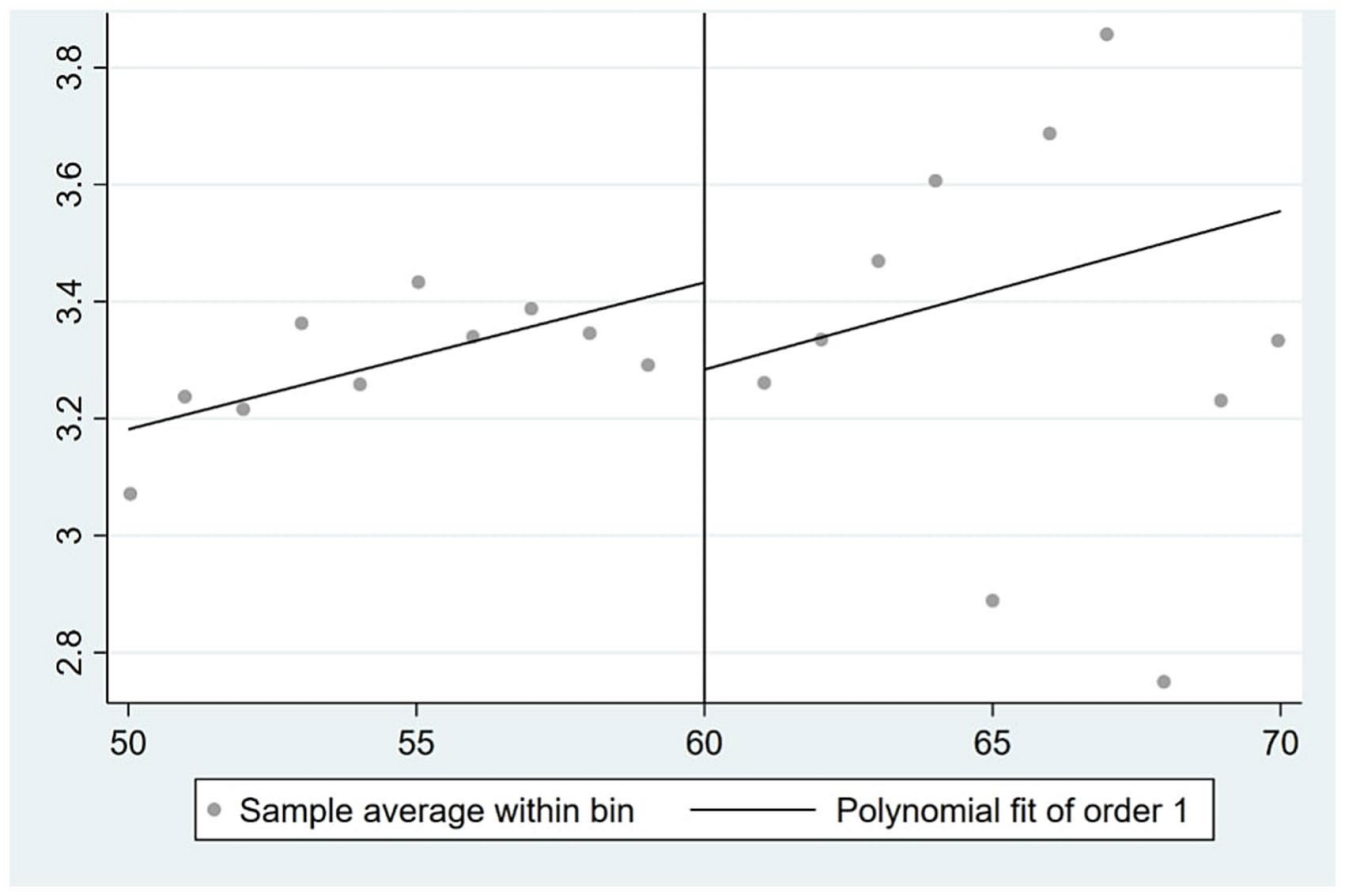
Effect of husband retirement on wife’s self-rated health.

**Figure 7:**
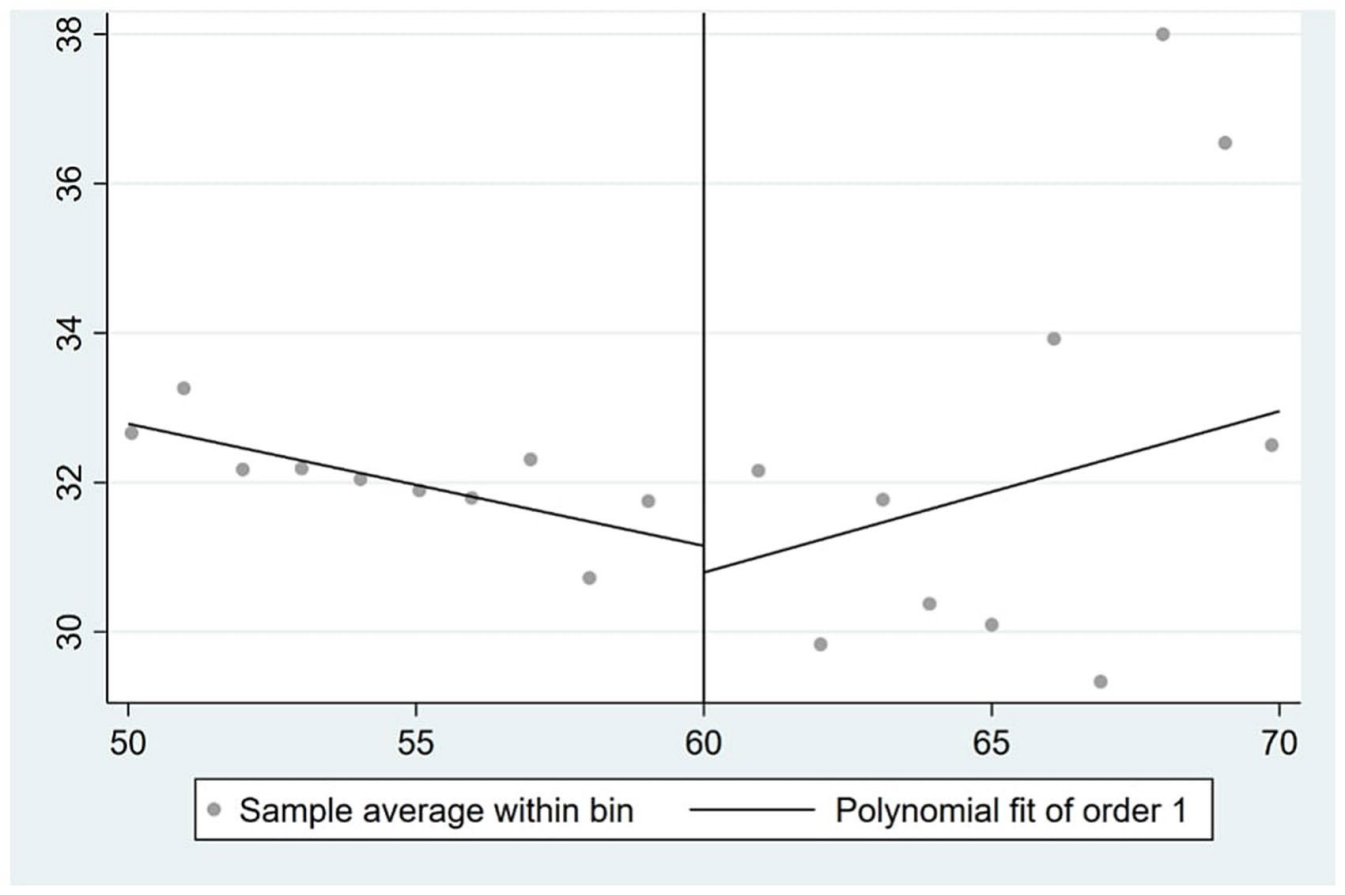
Effect of husband retirement on wife’s physical health.

**Figure 8:**
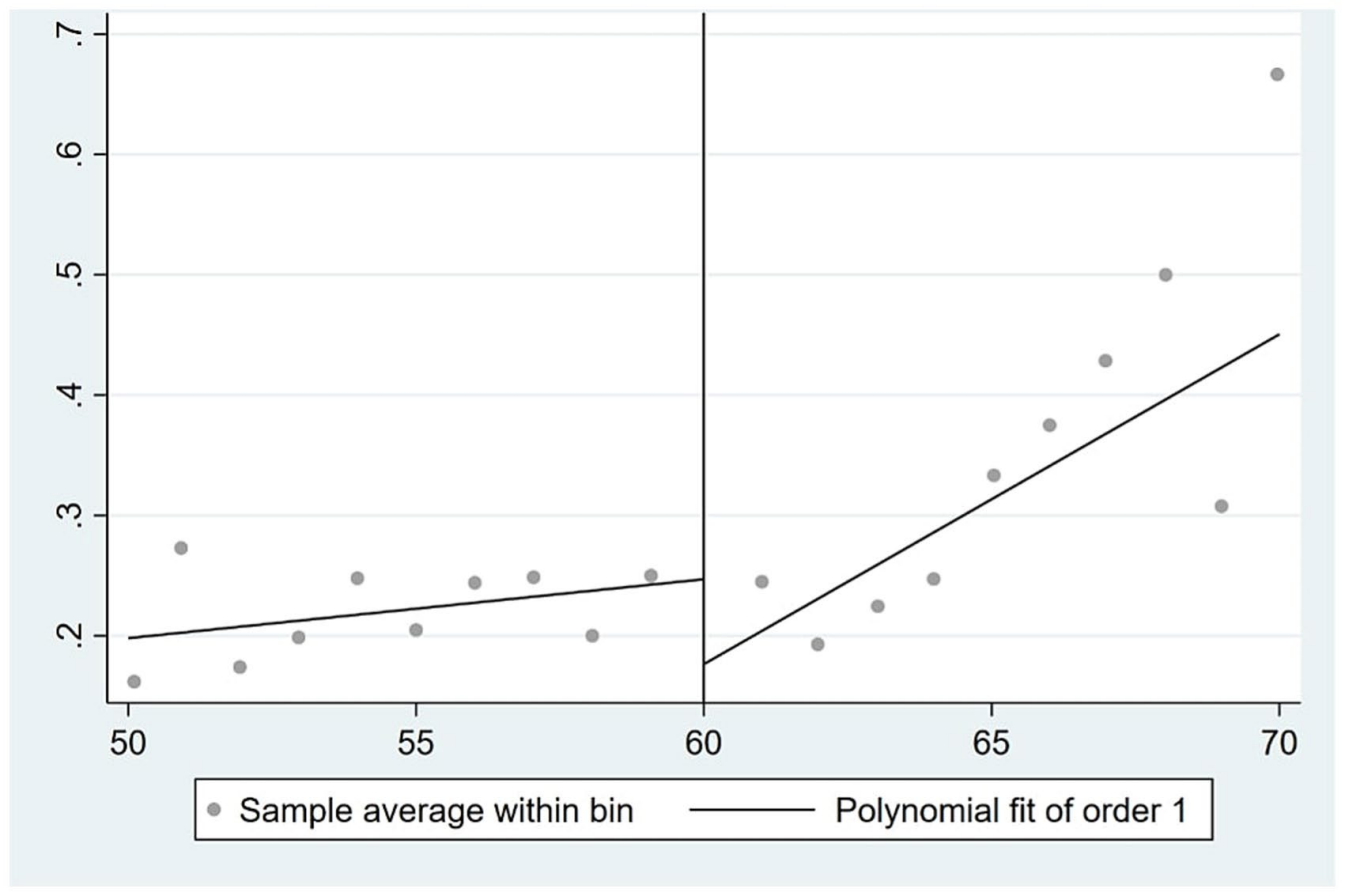
Effect of husband retirement on wife’s mental health.

Figures 6–8 depict the correlation between the age of the spouse and the self-rated health, physical health, and mental health of the wife, respectively. The retirement of the husband exerts a significant impact on the self-rated health and physical health of the wife, while demonstrating a modest effect on her mental health.

In this paper, the two-stage least squares method is used to further analyze the impact of husband’s retirement on the health of wives. Appendix Table A8 reports the results where columns (1), (2), and (3) exclude control variables, and columns (4), (5), and (6) include control variables. In terms of self-rated health, based on columns (1) and (4), there is gender heterogeneity in the health of spouses after retirement. Husband retirement has a more significant positive effect on the spouse’s self-rated health, with effect sizes ranging between 0.57 and 0.82. In terms of physical health, according to columns (2) and (5), neither husband nor wife retirement has a significant impact on the wife’s own physical health. Regarding mental health, based on columns (4) and (6), husband retirement does not have an impact on the wife’s mental health, and wife retirement does not have a significant effect on their own mental health. Overall, the husband’s retirement had no significant effect on the wife’s physical and mental health but contributed to the wife’s self-rated health at the 1% significance level.

Comparing and analyzing the impact of wife retirement on husband’s health, our findings are consistent with previous studies, indicating a clear gender heterogeneity in the health effects of spouse retirement. Specifically, when the wife retires, it does not significantly affect the husband’s self-rated health, but it does impact his physical and mental health. On the other hand, when the husband retires, it significantly improves the wife’s self-rated health, but does not have a significant impact on her physical health and mental health. More specifically, wife retirement significantly enhances the husband’s mental health, but significantly reduces his physical health. Conversely, husband retirement significantly improves the wife’s self-rated health. In terms of self-retirement, the husband’s retirement significantly reduces his own physical health, but does not have an impact on his self-rated health or mental health. Regardless of the health dimension, the wife’s retirement does not have a significant impact on her own health level. Therefore, it can be concluded that spouse retirement is more likely to affect an individual health level compared to their own retirement. Consequently, not considering the factor of spouse retirement may lead to inaccurate research results when examining the health impact of individual retirement.

In terms of control variables, the *hukou* status does not have a significant impact on the health of individuals. The likely reason for this is that the rural *hukou* individuals included in this study all participated in basic endowment insurance, enterprise supplementary endowment insurance, and endowment insurance for urban residents. The number of children mainly affects the health of men and not women. Academic qualifications can promote men’s self-rated health and women’s self-rated health and mental health.

## Robustness

### Continuity test for grouped variables versus control variables

To enhance the robustness of this study, the *rdcont* command in Stata was employed for testing. The *p*-values obtained are presented in Table 3, with estimated *p*-values of 0.717 for mate age and 0.612 for individual age. As a result, the null hypothesis cannot be rejected, indicating that the density function of spouse age and individual age is continuous at the cutoff. Therefore, it can be inferred that there is no artificial manipulation of age, and age is continuously distributed at the cutoffs.

**Table 3:** Continuity test of grouping variables.

	Spouse age	Individual age
*p*-value	0.717	0.612

The control variables selected in this paper are *hukou*, education and the quantity of children. In this paper, the continuity of the control variables is tested using the same two-step least squares method as mentioned earlier. The regression results in Appendix Table A9 indicate that both spouse retirement and individual retirement do not have a significant effect on the control variables. This suggests that the control variables are continuous with age and there is no treatment effect at the cutoff.

In conclusion, the grouping variables are not artificially controlled, and the control variables exhibit local smoothness. Therefore, this study utilizes fuzzy regression discontinuity to examine the impact of spouse retirement on individual health.

### Hetorogeneity analysis

#### Heterogeneity in individual retirement status

Chen posits that there exists a conjecture on the potential influence of disparities in individual retirement status on the health outcomes resulting from a spouse’s retirement (21). In order to investigate this matter, participants were categorized according to their retirement status, and the subsequent impact of a spouse’s retirement on an individual health was subsequently analyzed. The regression results pertaining to individual retirement status are presented in Appendix Table A10.

The data presented in columns (1) and (2) reveals significant variation in the impact of wife retirement on the health of husband. The retirement of the spouse has been found to positively impact the self-rated health, physical health, and mental health of the wife. Nevertheless, in cases when the husband has not yet retired, the retirement of the wife appears to have an adverse impact on the self-rated health, physical health, and mental health of the husband. Furthermore, the influence of a wife’s retirement has significant impacts solely on the health of husbands who have not yet retired.

In relation to columns (3) and (4), no discernible heterogeneity is observed with regards to the retirement status of the wife and her mental health. This implies that the retirement of the husband has a negative impact on the mental health of the wife. However, there is significant heterogeneity in terms of self-rated health and mental health based on the retirement status of the wife. In the case of retired wives, the retirement of the husband exacerbates their self-rated health and physical health. Conversely, for wives who are not retired, the retirement of the husband improves their self-rated health and physical health. Additionally, it is worth noting that the influence of the husband’s retirement on the self-rated health of wives who are not retired is statistically significant.

The influence of a spouse’s retirement on an individual health is contingent upon the individual’s own retirement status. Among the twelve possible combinations of three health dimensions and four individual retirement states, only two combinations demonstrate statistical significance. Firstly, when a wife retires, it negatively affects the physical health of her husband who remains employed. Secondly, when a husband retires, it positively impacts the self-rated health of his wife who remains employed. Consequently, it can be inferred that the health of individuals who have not retired is more vulnerable to the consequences of their spouse’s retirement, in comparison to those who have already retired.

### Mechanism

#### The mechanism hypothesis of spouse retirement on individual health

When analyzing the impact of spousal retirement on individual health, it is crucial to consider the mechanism of individual retirement. Hence, it is imperative to account for the individual’s retirement status as a control variable in order to examine the underlying mechanism of action. Based on the preceding investigation of diverse impacts of individual retirement status, it was determined that within the unretired cohort, the retirement of the husband exhibits a substantial decrease in the physical health of the wife, whereas the retirement of the wife demonstrates a significant enhancement in her self-rated health status. However, for the retired group, the influence of spouse retirement on individual health is not significant. Hence, this study centers its attention on the demographic subset of individuals who have not yet retired, with the aim of examining the impact of spousal retirement on the individual health.

Based on an extensive examination of existing literature and the incorporation of personal experiences, this study posits that the retirement of a spouse has the potential to impact an individual health through the modification of the couple’s overall life satisfaction and the subsequent alteration of each partner’s lifestyle choices. Furthermore, in this study, the CFPS questionnaire is employed to identify factors that serve as indicators of marital satisfaction, including pleasure with marital life, economic contribution to the spouse, and dwelling satisfaction. In addition, the study aims to examine the impact of retirement on the health of spouses by considering several variables such as the amount of time spent on housework, leisure activities, frequency of family dinners, sleep duration, exercise frequency, and other lifestyle variables.

#### The mechanism analysis of wife’s retirement on husband’s physical health

Based on the previously mentioned heterogeneity of individual retirement status, it has been established that the retirement of wives has a more significant impact on the physical health of husbands who are still working. Therefore, this study utilized the two-stage least squares method, as described earlier, to investigate the influence of wife retirement on the life satisfaction and lifestyle of husbands who have not yet retired. The dependent variables in this analysis were sleep time and exercise frequency. Although other potential mediating variables were also examined, it was determined that they were not suitable as mediating variables due to their limited capacity to affect levels of physical health, as evidenced by the regression results. By employing sleep time and exercise frequency as mediating variables, the study effectively controls for the influence of the husband’s own retirement on his health.

Based on the findings presented in Appendix Table A11, the retirement of wives has a significant impact on the sleep duration and exercise frequency of husbands who are not retired. However, it is important to note that these effects do not reach statistical significance. In order to investigate the potential mediating role of these variables, the mediation effect model previously described was employed for further analysis. Upon examining the results presented in Appendix Table A12, the coefficient of exercise frequency on the health of husbands is significantly positive. This suggests that an increase in exercise frequency is associated with a deterioration in husband’s health, which contradicts common knowledge. Furthermore, as the coefficient of sleep duration on husband health does not reach statistical significance, bootstrap analysis is required. The outcomes of the Stata procedure Bootstrap, as depicted in Appendix Table A13, provide evidence that the mediation effect of sleep duration is not statistically significant. This is supported by the fact that the confidence interval of sleep duration encompasses the value of zero.

Hence, the present study did not identify any mediating variables that could account for the impact of a wife’s retirement on the physical health of her husband. This phenomenon could perhaps be attributed to the existence of other mechanisms that are influencing the situation. Combined with the actual living situation, the wife has a lot of leisure time after retirement, and may be more willing to improve her cooking skills, and because work occupies attention, the non-retired husband will pay less attention to physical health than the retired husband, and the non-retired husband will consume more in the diet, which will increase the incidence of chronic diseases such as hypertension and diabetes, and worsen the physical health. However, the identification of acceptable mediating variables was not possible due to deficiencies in the CFPS data. It is plausible that the impact of a wife’s retirement on the physical health of husbands is less substantial when compared to the influence of a husband’s retirement on the self-rated health of wives, which exhibits statistical significance at a 10% level.

#### The mechanism analysis of husband’s retirement on wife’s self-rated health

The influence of husbands’ retirement on the self-rated health of unretired women is more pronounced than that on retired spouses, as shown by the heterogeneity observed in individual retirement status. Consequently, this research employed the 2SLS to investigate the impact of husband’s retirement on the marital satisfaction and lifestyle of unretired women, with each mediating variable serving as the dependent variable. This methodology successfully mitigates the influence of the wife’s retirement on her self-rated health.

Based on the findings presented in Appendix Table A14, the retirement of husbands has a significant positive impact on various aspects of marital life for unretired wives. Specifically, their satisfaction with marital life, satisfaction with the spouse’s economic contribution, the amount of time allocated to watching TV and movies, as well as sleep duration, all experience substantial improvements. However, it is worth noting that the wife’s economic contribution to the spouse and the frequency of family dinners exhibited a great but noisy increase. Similarly, the proportion of housework and the frequency of exercise both decreased, but these changes were also not statistically significant.

Judging from empirical observations, it is plausible to surmise that the underlying rationale behind these findings lies in the phenomenon whereby husbands, subsequent to retirement, augment their commitment to familial responsibilities, so affording wives an increased opportunity for leisure. Consequently, this dynamic substantially enhances the overall life satisfaction of wives.

In order to ascertain the extent to which these mediating variables contribute to the enhancement of spouses’ self-rated health, additional research was undertaken with the previously proposed mediation effect model.

Based on the coefficients and significance presented in the first row of Appendix Table A15, it can be inferred that there exists a significant positive relationship between wives’ satisfaction with marital life, satisfaction with the spouse’s economic contribution, satisfaction with the spouse’s household contribution, and an increase in sleep time with their self-rated health levels. Furthermore, a decrease in housework is also found to have a positive impact on wives’ self-rated health, while the number of exercises does not exhibit a significant effect on their self-rated health, thus corroborating our hypothesis.

Based on the findings presented in the first row of Appendix Table A14, the three mediating variables (marital satisfaction, satisfaction with spouse’s household contribution, and sleep time) witnessed a significant improvement. This suggests that these variables play a role in the process by which husband retirement influences the self-rated health of wives. However, the coefficient of spouse retirement did not yield a significant result for the variables of satisfaction with spouse’s economic contribution and household proportion. Hence, the researchers utilized the Bootstrap approach to further ascertain the presence of a mediation effect. The findings obtained from the Stata procedure Bootstrap, as presented in Appendix Table A16, reveal that the confidence intervals pertaining to satisfaction with spouse’s economic contribution exclude the value of 0. This observation implies that satisfaction with spouse’s economic contribution can potentially serve as a mediating variable in elucidating the impact of husband retirement on wives’ self-rated health. However, it should be noted that the confidence interval for the household proportion encompasses the value of 0, suggesting that it lacks the characteristics necessary to be classified as a mediating variable.

In summary, the retirement of a husband has a substantial impact on various aspects of marital satisfaction, including contentment with the spouse’s financial and domestic contributions, as well as sleep duration. Consequently, this improvement in marital dynamics contributes to an enhanced level of self-rated health among wives who have not yet retired.

Hence, the primary argument posited in this article asserts that the retirement of a spouse might really result in variations to an individual’s marital satisfaction and sleep duration, ultimately impacting their overall health. The retirement of the husband has a significant effect on the marital satisfaction of wives who have not yet retired, leading to a substantial rise in their sleep duration and consequently enhancing their self-rated health to a large degree.

## Discussion

The study found that there is gender heterogeneity in the impact of spouse retirement on individual health. Specifically, wife retirement has a significant negative effect on the husband’s mental health, while husband retirement has a significant impact on the wife’s self-rated health but not on her physical health and mental health (16). Although consistent with previous articles (21, 22), the effects of spousal retirement on individual health show gender heterogeneity, our article explicitly delineates the three dimensions of health, which allows us to clarify in which dimensions of health gender heterogeneity appears. For the dimension of self-assessed health, because the main sample is from urban China, we believe that people have a more accurate perception of their own health, and the degree of bias will be small. Our results suggest a relatively significant difference in the impact of spousal retirement due to gender in terms of mental health and self-rated health. Why these two dimensions of health is possible because of the differences between male and female in personality, self-perception, and social status, which requires more research to explore and confirm. Gender heterogeneity is also demonstrated in another way. Male retirement can have a detrimental effect on physical health but does not significantly impact self-rated health and mental health. Female retirement does not have a significant impact on their own health. Obviously, the impact of spousal retirement on an individual’s health is more powerful. The study suggests that when examining the impact of retirement on health, it is important to consider not only individual retirement but also the impact of spouse retirement. Control variables such as academic degree does not significantly affect individual health, and the number of children mainly affects the physical and mental health of male but not female. Education can promote self-rated health and mental health for both male and female.

Based on these findings and the underlying theoretical framework, we propose the following policy recommendations. Firstly, delayed retirement policies should balance economic needs with public health by supporting the well-being of both partners. Secondly, implement retirement reforms progressively to minimize adverse health effects. Policies should be adaptable, considering factors such as gender, occupation, region, income, and spousal retirement status. Flexible approaches, like allowing certain professions to opt out of mandatory retirement or enabling reduced work hours for older adults, can enhance policy effectiveness and individual well-being. Lastly, enhance the health of middle-aged and elderly populations through community-based activities, improving living environments, and fostering harmonious marital relationships. Encouraging shared household responsibilities and providing support for marital satisfaction can reduce stress and improve overall health outcomes.

## Conclusion

The main findings of this study are that wife retirement has a significant negative impact on the husband’s physical health and positive effect on the husband’s mental health, while husband retirement has a significant positive influence on the wife’s self-rated health but not on her physical health and mental health. After dividing the different retirement statuses of individuals, the study found that the unretired group is more influenced by the retirement status of their spouse compared to the retired group. Specifically, wife retirement significantly reduces the physical health level of unretired husbands, while the effect on the health of retired husbands is not significant. Husband retirement significantly improves the self-rated health level of unretired wives, while the effect on the health of retired wives is not significant. Based on the above findings, we should focus on the health of our husbands when their wives retire, especially for the unretired husband, and the husbands should stick to their previous lifestyles and not change too dramatically. We also studied the impact of their own retirement on their individual health. The analysis conducted in the discussion suggests that the impact of a spouse’s retirement on health is more substantial than the impact of one’s own retirement on health, which makes a huge contribution to defining research directions for future studies, and researchers cannot ignore the influence of spouses when studying the relationship between retirement and health.

The study found that marital satisfaction and sleep time are likely mechanisms through which spouse retirement affects individual health. For unretired wives, husband retirement significantly improves marital satisfaction, satisfaction with the spouse’s economic contribution, satisfaction with the spouse’s household contribution, and sleep time, leading to a significant improvement in self-rated health. The discovery of this mechanism is one of the contributions of this paper, and correspondingly, we suggest that middle-aged and elderly husbands should be actively involved in household chores, which will help their wives to have more sleep time and rest time, which will also greatly enhance marital satisfaction of both spouses. Community and street personnel can play a role in facilitating this communication and understanding. These include improving the lifestyle of the elderly population by increasing exercise frequency and recreational activities, creating a harmonious community environment by reducing noise and light pollution, and improving marital satisfaction to enhance health levels through effective communication and understanding between couples experiencing family conflicts.

Our results provide evidence of the impact of spousal retirement on individual health, consequently, the government should take into account the impact of retirement on health when implementing a policy of postponing the retirement or formulating a retirement policy. First, according to the significant positive effect of husband’s retirement on wife’s self-assessed health and the significant negative effect on her own health, when delaying men’s retirement, both women’s health and men’s health are at threat, thus increasing the cost of pension. Since females retire ten years earlier than males, husbands generally do not retire when their wives do. As wife’s retirement has a significant adverse effect on the health of the non-retired husband, the retirement age of Chinese women can be raised appropriately. There are two limitations in this study. First, based on the available data, our study only found the mechanism of husband’s retirement on wife’s impact, while the mechanism of wife’s retirement on husband’s health is not obvious for the time being. Second, because of the characteristics of the fuzzy regression discontinuity, the effect of spouses’ retirement on health found in this study is short-term, and the long-term effect needs to be replaced with a suitable model for further research and analysis.

## Data Availability

Publicly available datasets were analyzed in this study. This data can be found at: https://www.isss.pku.edu.cn/cfps/en/.

## Footnotes

^1^See the official report of Ministry of Civil Affairs of the People’s Republic of China for details, https://www.mca.gov.cn/n156/n2679/c1662004999980001751/attr/360830.pdf.
