# Supplementary material for: The impact of spousal retirement on health—an empirical analysis based on CFPS
Source: Front Med (Lausanne). 2025 Jan 24;11:1518936. doi: 10.3389/fmed.2024.1518936 (PMC11803584; doi:10.3389/fmed.2024.1518936)
Supplement: Supplementary file 1 [file Data_Sheet_1.docx]

Appendix A

**Table A1. Survey questions for CES-D8 and CES-D20.**

| **Order number** | **CES-D8** | **CES-D20** | |
| --- | --- | --- | --- |
| 1 | I feel depressed | I am troubled by some little things | My sleep is not good |
| 2 | I find it very hard to do anything | I don't want to eat. I have a bad appetite | I feel happy |
| 3 | My sleep is not good | I feel frustrated, even having the help from family and friends doesn't work | I speak less than usual |
| 4 | I feel happy | I think I'm no worse than the others | I feel lonely |
| 5 | I feel lonely | I have had great difficulty concentrating on doing things | I think people are unfriendly to me |
| 6 | I live a happy life | I feel depressed | I live a happy life |
| 7 | I feel sad | I find it very hard to do anything | I cried or wanted to cry |
| 8 | I don't think life can go on | I have great hope for the future | I feel sad |
| 9 | - | I think it's always been a failure | I don't think other people like me |
| 10 | - | I'm afraid | I don't think life can go on |

**Table A2. Variable description.**

|  | **Variable name** | **Concrete issue** | **Span** |
| --- | --- | --- | --- |
| Dependent variable | Self-rated health | How good is your own health | Very healthy=1, unhealthy =5 |
|  | Physical health | Chronic disease within half a year | No =0, Yes =1 |
|  | Mental health | CES-D20 | 20-80 |
| Independent variable | Spouse retirement status | What is the main reason for not working at present | Non-retirement=0, retired =1 |
| Control variable | Personal retirement status | What is the main reason for not working at present | Non-retirement=0, retired =1 |
|  | Age | Judging by the date of birth | 40 – 60 for males and 50 – 70 for females |
|  | Spouse age | Judging by the date of birth | 40 – 60 for males and 50 – 70 for females |
|  | *Hukou* | *Hukou* status | Rural *hukou* =0, non-rural *hukou* =1, resident *hukou* =2 |
|  | Education | Education situation | Illiterate / semi-illiterate ==1, PhD =8 |
|  | Quantity of children | According to the child code calculation | 0-10 |
| Mediating variable | Satisfaction of marital life | How satisfied are you with your current marriage / cohabitation " life | Very dissatisfied=1, very satisfied =5 |
|  | Satisfaction with the spouse's economic contribution | How satisfied are you with your partner's financial contribution to the family | Very dissatisfied=1, very satisfied =5 |
|  | Satisfaction with the spouse's household contribution | How satisfied are you with your partner's contribution to the household | Very dissatisfied=1, very satisfied =5 |
|  | The proportion of housework | Based on the couple's housework time calculation | 0-1 |
|  | The time spend watching TV or movies | Each week is about hours of TV, movies and other video shows | 0-77 |
|  | Dinner times with family | How many nights a week do you eat with your family | 0-7 |
|  | Sleep time | Calculated according to the questionnaire | 2-12 |
|  | The number of times you exercise | How many times have you exercised in the past week | 0-21 |

**Table A3. Descriptive statistics of the female sample.**

|  |  | (1) | (2) | (3) | (4) | (5) | (6) | (7) | (8) | (9) | | (10) |
| --- | --- | --- | --- | --- | --- | --- | --- | --- | --- | --- | --- | --- |
|  |  | Husband unretired | | | | | Husband retired | | | | | |
| Variable species | Variable name | Sample capacity | Average value | Standard deviation | Min | Max | Sample capacity | Average value | Standard deviation | Min | | Max |
| Dependent variables | Self-rated health | 4,767 | 3.292 | 1.069 | 1 | 5 | 789 | 3.317 | 1.063 | 1 | 5 | |
|  | Physical health | 4,767 | 0.778 | 0.415 | 0 | 1 | 789 | 0.745 | 0.436 | 0 | 1 | |
|  | Mental health | 3,325 | 32.168 | 7.507 | 21 | 68 | 483 | 31.006 | 6.593 | 22 | 54 | |
| Control variables | Personal retirement status | 4,767 | 0.207 | 0.405 | 0 | 1 | 789 | 0.669 | 0.471 | 0 | 1 | |
|  | Age | 4,767 | 52.78 | 4.184 | 40 | 60 | 789 | 57.07 | 3.079 | 44 | 60 | |
|  | Spouse age | 4,767 | 54.96 | 3.688 | 50 | 70 | 789 | 60.67 | 3.205 | 51 | 70 | |
|  | *Hukou* | 4,767 | 1.007 | 0.368 | 0 | 2 | 789 | 1.062 | 0.266 | 0 | 2 | |
|  | Education | 4,709 | 3.232 | 1.362 | 1 | 9 | 769 | 3.234 | 1.193 | 1 | 9 | |
|  | Quantity of children | 4,767 | 1.268 | 0.696 | 0 | 9 | 789 | 0.957 | 0.616 | 0 | 3 | |
| Mediating variables | Satisfaction of marital life | 3,475 | 4.369 | 0.920 | -1 | 5 | 575 | 4.504 | 0.803 | 1 | 5 | |
|  | Satisfaction with the spouse's economic contribution | 3,475 | 4.141 | 1.044 | 1 | 5 | 575 | 4.381 | 0.961 | 1 | 5 | |
|  | Satisfaction with the spouse's household contribution | 3,475 | 3.869 | 1.214 | 1 | 5 | 575 | 4.167 | 1.106 | 1 | 5 | |
|  | The proportion of housework | 4,749 | 0.697 | 0.241 | 0 | 1 | 789 | 0.637 | 0.232 | 0 | 1 | |

**Table A4. Two-stage least squares stage I estimation results for females.**

|  | (1) | (2) | (3) | (4) |
| --- | --- | --- | --- | --- |
| Female sample | Individual retirement | Husband retirement | Individual retirement | Husband retirement |
| Whether individuals exceed the legal retirement age $D_{i}$ | 0.126*** |  | 0.124*** |  |
|  | (8.72) |  | (8.29) |  |
| Whether the spouse exceeds the legal retirement age $D_{i}^{p}$ |  | 0.241*** |  | 0.238*** |
|  |  | (11.97) |  | (11.84) |
| *Hukou* |  |  | 0.089*** | 0.009** |
|  |  |  | (6.91) | (2.54) |
| Education |  |  | 0.033*** | 0.012*** |
|  |  |  | (8.03) | (3.65) |
| quantity of children |  |  | -0.091*** | -0.050*** |
|  |  |  | (-11.76) | (-8.88) |
| The difference between the age and the legal retirement age | 0.021*** |  | 0.021*** |  |
|  | (15.92) |  | (14.41) |  |
| $D_{i}$*（The difference between the age and the legal retirement age） | 0.028*** |  | 0.024*** |  |
|  | (10.44) |  | (8.20) |  |
| The difference between the spouse's age and the legal retirement age |  | 0.241*** |  | 0.032*** |
|  |  | (11.97) |  | (9.10) |
| $D_{i}^{p}$*（The difference between the spouse's age and the legal retirement age） |  | 0.032*** |  | 0.028*** |
|  |  | (9.25) |  | (4.02) |
| Constant | 0.068*** | 0.173*** | -0.014 | 0.162*** |
|  | (5.07) | (13.04) | (-0.58) | (7.70) |
| Sample capacity | 5556 | 5556 | 5478 | 5482 |
| F value | 329.995 | 209.450 | 203.148 | 145.996 |

**Table A5. Results of two-stage least squares stage I estimation for the full sample.**

|  | (1) | (2) | (3) | (4) |
| --- | --- | --- | --- | --- |
| Full sample | Individual retirement | Spouse retirement | Individual retirement | Spouse retirement |
| Whether individuals exceed the legal retirement age $D_{i}$ | 0.145*** |  | 0.146*** |  |
|  | (11.99) |  | (12.19) |  |
| Whether the spouse exceeds the legal retirement age $D_{i}^{p}$ |  | 0.145*** |  | 0.147*** |
|  |  | (12.06) |  | (12.31) |
| *Hukou* |  |  | 0.067*** | 0.015*** |
|  |  |  | (8.44) | (4.72) |
| Education |  |  | 0.022*** | 0.023*** |
|  |  |  | (7.99) | (8.28) |
| quantity of children |  |  | -0.076*** | -0.077*** |
|  |  |  | (-15.79) | (-15.96) |
| The difference between the age and the legal retirement age | 0.019*** |  | 0.018*** |  |
|  | (17.26) |  | (16.80) |  |
| $D_{i}$*（The difference between the age and the legal retirement age） | 0.011*** |  | 0.009*** |  |
|  | (5.21) |  | (4.43) |  |
| The difference between the spouse's age and the legal retirement age |  | 0.145*** |  | 0.018*** |
|  |  | (12.06) |  | (16.60) |
| $D_{i}^{p}$*（The difference between the spouse's age and the legal retirement age） |  | 0.018*** |  | 0.009*** |
|  |  | (16.97) |  | (4.43) |
| Constant | 0.144*** | 0.140*** | 0.094*** | 0.109*** |
|  | (14.77) | (14.42) | (5.81) | (6.39) |
| Sample capacity | 11120 | 11120 | 10968 | 10968 |
| F value | 513.016 | 503.777 | 348.766 | 342.607 |

**Table A6. Results of two-stage least squares stage II estimation for the full** **sample.**

|  | (1) | (2) | (3) | (4) | (5) | (6) |
| --- | --- | --- | --- | --- | --- | --- |
| All samples | Self-rated | Physical | Mental | Self-rated | Physical | Mental |
| Retirement | 0.156 | 0.140 | -3.161 | 0.035 | 0.174* | -3.904* |
|  | (0.56) | (1.37) | (-1.37) | (0.13) | (1.72) | (-1.70) |
| Spouse retirement | -0.226 | 0.220** | 3.761* | -0.693*** | 0.203** | -0.720 |
|  | (-0.83) | (2.12) | (1.67) | (-3.20) | (2.50) | (-0.41) |
| *Hukou* | - | - | - | 0.042 | -0.030** | 0.258 |
|  | - | - | - | (1.10) | (-2.12) | (0.87) |
| Education | - | - | - | -0.031*** | -0.002 | -0.296*** |
|  | - | - | - | (-2.76) | (-0.58) | (-3.11) |
| Quantity of children | - | - | - | -0.089*** | 0.027** | -0.287 |
|  | - | - | - | (-2.62) | (2.12) | (-1.04) |
| The difference between the age and the legal retirement age | 0.017** | 0.003 | 0.170** | 0.018** | 0.002 | 0.181*** |
|  | (2.05) | (0.85) | (2.51) | (2.31) | (0.62) | (2.73) |
| $D_{i}$* (Difference between age and legal retirement age) | -0.011 | 0.011*** | 0.074 | -0.014* | 0.012*** | 0.077 |
|  | (-1.36) | (3.43) | (1.08) | (-1.69) | (3.68) | (1.11) |
| The difference between the spouse's age and the legal retirement age | 0.004 | -0.004 | -0.261*** | 0.015** | -0.003 | -0.138*** |
|  | (0.46) | (-1.27) | (-3.97) | (2.40) | (-1.31) | (-2.64) |
| $D_{i}^{p}$* (Difference between spouse age and legal retirement age) | -0.006 | 0.007** | 0.054 | -0.008 | 0.008** | 0.077 |
|  | (-0.71) | (2.12) | (0.78) | (-0.94) | (2.35) | (1.10) |
| Constant | 3.248*** | 0.895*** | 30.206*** | 3.455*** | 0.884*** | 31.345*** |
|  | (29.05) | (21.49) | (35.67) | (26.51) | (18.63) | (31.31) |
| Sample capacity | 11120 | 11120 | 7618 | 10892 | 10892 | 7390 |

**Table A7. Results of two-stage least squares stage II estimation of the male sample.**

|  | (1) | (2) | (3) | (4) | (5) | (6) |
| --- | --- | --- | --- | --- | --- | --- |
| Male | Self-rated | Physical | Mental | Self-rated | Physical | Mental |
| Retirement | 0.255 | 0.152* | 0.401 | 0.202 | 0.153* | -1.842 |
|  | (1.10) | (1.74) | (0.21) | (0.84) | (1.71) | (-0.94) |
| Spouse retirement | -0.197 | 0.224** | -5.716** | -0.175 | 0.256** | -7.315*** |
|  | (-0.70) | (2.11) | (-2.48) | (-0.62) | (2.44) | (-3.12) |
| *Hukou* | - | - | - | 0.021 | -0.015 | 0.277 |
|  | - | - | - | (0.46) | (-0.87) | (0.82) |
| Education | - | - | - | -0.027** | 0.001 | 0.055 |
|  | - | - | - | (-1.96) | (0.26) | (0.48) |
| Quantity of children | - | - | - | -0.051 | 0.025* | -0.568* |
|  | - | - | - | (-1.31) | (1.75) | (-1.84) |
| The difference between the age and the legal retirement age | 0.026** | -0.003 | -0.018 | 0.020 | 0.007 | 0.165 |
|  | (2.40) | (-0.86) | (-0.21) | (1.44) | (1.22) | (1.36) |
| $D_{i}$* (Difference between age and legal retirement age) | -0.035** | 0.013** | 0.083 | -0.021 | 0.020** | 0.324* |
|  | (-2.51) | (2.17) | (0.75) | (-1.18) | (2.54) | (1.95) |
| The difference between the spouse's age and the legal retirement age | -0.006 | -0.004 | 0.225** | -0.004 | -0.002 | 0.247*** |
|  | (-0.61) | (-1.01) | (2.51) | (-0.34) | (-0.53) | (2.75) |
| $D_{i}^{p}$* (Difference between spouse age and legal retirement age) | -0.000 | 0.001 | -0.245*** | -0.027** | -0.016*** | -0.112 |
|  | (-0.05) | (0.36) | (-5.24) | (-2.05) | (-3.36) | (-1.05) |
| Constant | 3.386*** | 0.099** | 30.985*** | 3.455*** | 0.116*** | 31.345*** |
|  | (26.34) | (2.08) | (31.42) | (26.51) | (2.44) | (31.31) |
| Sample capacity | 5564 | 5564 | 3810 | 5450 | 5450 | 3696 |

**Table A8. Results of two-stage least squares stage II estimation of the female sample.**

|  | (1) | (2) | (3) | (4) | (5) | (6) |
| --- | --- | --- | --- | --- | --- | --- |
| Female | Self-rated | Physical | Mental | Self-rated | Physical | Mental |
| Retirement | 0.284 | 0.045 | -3.245 | 0.201 | 0.138 | 0.066 |
|  | (0.51) | (0.23) | (-0.69) | (0.35) | (0.70) | (0.01) |
| Spouse retirement | -0.576** | -0.061 | 1.620 | -0.815*** | -0.084 | 1.149 |
|  | (-2.52) | (-0.66) | (0.83) | (-3.60) | (-0.94) | (0.59) |
| *Hukou* |  |  |  | 0.024 | -0.035 | -0.093 |
|  |  |  |  | (0.34) | (-1.39) | (-0.16) |
| Education |  |  |  | -0.046** | -0.005 | -0.657*** |
|  |  |  |  | (-1.98) | (-0.54) | (-3.22) |
| Quantity of children |  |  |  | -0.052 | 0.008 | -0.102 |
|  |  |  |  | (-0.87) | (0.40) | (-0.21) |
| The difference between the age and the legal retirement age | 0.008 | 0.008 | 0.027 | 0.010 | 0.005 | -0.085 |
|  | (0.40) | (1.22) | (0.17) | (0.51) | (0.80) | (-0.51) |
| $D_{i}$* (Difference between age and legal retirement age) | 0.003 | 0.013** | 0.161 | 0.006 | 0.013*** | 0.168 |
|  | (0.21) | (2.45) | (1.40) | (0.41) | (2.65) | (1.51) |
| The difference between the spouse's age and the legal retirement age | 0.035*** | 0.006 | -0.085 | 0.044*** | 0.008 | -0.062 |
|  | (2.58) | (1.02) | (-0.72) | (3.29) | (1.37) | (-0.53) |
| $D_{i}^{p}$* (Difference between spouse age and legal retirement age) | 0.036** | 0.024*** | 0.107 | 0.042** | 0.027*** | 0.173 |
|  | (2.01) | (3.04) | (0.72) | (2.32) | (3.42) | (1.14) |
| constant | 3.304*** | 0.850*** | 31.347*** | 3.563*** | 0.827*** | 33.170*** |
|  | (23.51) | (16.54) | (28.24) | (27.33) | (17.10) | (31.98) |
| Sample capacity | 5556 | 5556 | 3808 | 5442 | 5442 | 3694 |

**Table A9. Continuity tests for control variables.**

|  | (1) | (2) | (3) |
| --- | --- | --- | --- |
| Control variable | *Hukou* | Education | Quantity of children |
| Retirement | 0.120 | 0.048 | -0.019 |
|  | (1.42) | (0.15) | (-0.11) |
| Spouse retirement | 0.072 | 0.073 | 0.026 |
|  | (0.88) | (0.22) | (0.15) |
| Vintage virtual variable | true | true | true |
| Sample capacity | 11120 | 10968 | 11120 |

**Table A10. Influence of spouse's retirement on individual health under different retirement status.**

|  | (1) | (2) | (3) | (4) |
| --- | --- | --- | --- | --- |
|  | Male | | Female | |
|  | Retired husband | Unretired husband | Retired wife | Unretired wife |
| Self-rated health | -1.041 | 0.571 | 0.037 | -1.984** |
|  | (-0.76) | (1.08) | (0.15) | (-2.28) |
| Sample capacity | 775 | 4675 | 1481 | 3961 |
| Physical health | -0.930 | 0.314* | 0.093 | -0.454 |
|  | (-1.28) | (1.66) | 0.079 | (-1.46) |
| Sample capacity | 775 | 4675 | (0.77) | 3961 |
| Mental health | -20.998 | 0.090 | 1.467 | 5.921 |
|  | (-0.89) | (0.02) | (0.57) | (1.04) |
| Sample capacity | 461 | 3235 | 936 | 2758 |

**Table A11. Effect of wife retirement on sleeping time and number of exercises of unretired husbands.**

|  | (1) | (2) |
| --- | --- | --- |
| Male | Sleep time | Exercise times |
| Nonretirement | -0.962 | -0.342 |
|  | (-1.54) | (-0.23) |
| Sample capacity | 4699 | 4705 |

**Table A12. Effects of sleep time and exercise times on the health of unretired husband.**

|  | （1） | （2） |
| --- | --- | --- |
| Male | Sleep time | Exercise times |
| Nonretirement | -0.007 | 0.005** |
|  | (-1.38) | (2.43) |
| The effect of husband's retirement on wife's health | 0.304 | 0.316* |
|  | (1.60) | (1.67) |
| Whether there is a mediation effect | Further testing using bootstrap is required | non-existent |
| Sample capacity | 4699 | 4705 |

**Table A13. Results of the bootstrap test.**

|  | (1) | (2) | (3) | (4) | (5) |
| --- | --- | --- | --- | --- | --- |
| Indigo effect | Coefficient | Results of the Bootstrap-test | Z value | P value | Confidence interval |
| Sleep time | 0.0054 | 0.0063 | 0.87 | 0.384 | （-0.0068，0.0177） |

**Table A14. Effects of husband's retirement on life satisfaction and lifestyle of non-retired wives.**

|  | (1) | (2) | (3) | (4) | (5) | (6) | (7) | (8) |
| --- | --- | --- | --- | --- | --- | --- | --- | --- |
| Female | Marriage satisfaction | Satisfaction with the spouse's economic contribution | Satisfaction with the spouse's household contribution | The proportion of housework | How much time you spend watching TV or movies | Have dinner with your family how many times | Sleep time | Exercise times |
| Whether the husband is retired | 1.834** | 1.272 | 1.827* | -0.041 | 16.647** | 1.604 | 2.255** | -1.891 |
|  | (2.26) | (1.50) | (1.92) | (-0.25) | (1.98) | (1.46) | (2.16) | (-0.84) |
| Sample capacity | 2955 | 2955 | 2955 | 3977 | 3993 | 3993 | 3993 | 3979 |

**Table A15. Effects of marital life satisfaction and lifestyle on self-rated health of non-retired wives.**

|  | (1) | (2) | (3) | (4) | (5) | (6) | (7) | (8) |
| --- | --- | --- | --- | --- | --- | --- | --- | --- |
| Female | Marriage satisfaction | Satisfaction with the spouse's economic contribution | Satisfaction with the spouse's household contribution | The proportion of housework | How much time you spend watching TV or movies | Have dinner with your family how many times | Sleep time | Exercise times |
| The effect of mediator variables on self-rated health | -0.105*** | -0.070*** | -0.054*** | -0.076 | 0.004** | 0.002 | -0.043*** | 0.001 |
|  | (-5.04) | (-3.62) | (-2.80) | (-0.80) | (2.26) | (0.21) | (-2.86) | (0.14) |
| Effect of husband retirement on wife self-rated health | -1.159 | -1.263 | -1.254 | -2.052** | -2.049** | -1.988** | -1.901** | -1.982** |
|  | (-1.37) | (-1.46) | (-1.43) | (-2.34) | (-2.34) | (-2.29) | (-2.24) | (-2.30) |
| Whether there is a mediation effect | existence | Further judgment is required | existence | Further judgment is required | inexistence | inexistence | existence | inexistence |
| Sample capacity | 2955 | 2955 | 2955 | 3977 | 3993 | 3993 | 3993 | 3979 |

**Table A16. Results of the bootstrap test.**

|  | (1) | (2) | (3) | (4) | （5) |
| --- | --- | --- | --- | --- | --- |
| Indigo effect | Coefficient | Bootstrap Test the results | Z value | P value | Confidence interval |
| Spouse economic contribution degree | -1.2848 | 0.3442 | -0.363 | 0.000 | (-1.95951，-0.61011) |
| The proportion of housework | 0.40528 | 0.3765 | 1.08 | 0.282 | (-0.33266, 1.1432 |
